# Prevalence and Recurrence Rates of Cytomegalovirus Infection Among Patients With Hematological Diseases in the Western Brazilian Amazon: A Cross-Sectional Study

**DOI:** 10.3389/fpubh.2021.692226

**Published:** 2021-10-07

**Authors:** Jean de Melo Silva, Renato Pinheiro-Silva, Regiane Costa de Oliveira, Carlos Eduardo de Castro Alves, Anderson Nogueira Barbosa, Gemilson Soares Pontes

**Affiliations:** ^1^Programa de Pós-graduação em Ciências Hematológicas, Universidade do Estado do Amazonas, Manaus, Brazil; ^2^Programa de Pós-graduação em Imunologia Básica e Aplicada, Universidade Federal do Amazonas, Manaus, Brazil; ^3^Laboratório de Virologia e Imunologia, Instituto Nacional de Pesquisa da Amazônia, Manaus, Brazil

**Keywords:** cytomegalovirus, prevalence, epidemiology, hematological diseases, recurrent infection, Brazilian Amazon

## Abstract

Cytomegalovirus (CMV) is a worldwide distributed pathogen that may cause serious complications in patients with hematological diseases. This study aimed to serologically characterize CMV infection in patients suffering from hematological diseases in Amazonas state, Brazil. Serum samples from 323 patients were tested for the presence of anti-CMV IgM or IgG antibodies using an enzyme-linked immunosorbent assay. Positive samples for IgM were submitted to the IgG avidity test to differentiate primary infection from recurrent infection. An epidemiological questionnaire was administered to collect the sociodemographic information of the study population. The overall prevalence of CMV infection verified in this study was 91.3%. The highest rates were found in patients suffering from platelet disorders (94.5%), anemia (93.3%), or leukemia (91%). The study population was predominantly composed of individuals with low socioeconomic status. Blood transfusions were more common in patients with anemia or leukemia, but this variable was not correlated with the seropositivity for CMV infection. Measurement of IgG avidity in patients positive for anti-CMV IgM demonstrated a recurrent infection rate of 5.2% (17/323). Over 80% of recurrent infections occurred in patients with acute lymphocytic leukemia (ALL) or anemia. Our findings indicated that CMV infection is highly prevalent in patients from the western Brazilian Amazon who have hematological diseases. The prevalence observed progressively rose with increasing age, whereas anemia or ALL figured as risk factors for the recurrence of CMV infection.

## Introduction

Cytomegalovirus (CMV) is a human herpes virus that is endemic throughout the world (1). Viral transmission occurs via intimate contact with infected bodily fluids and through transplacental transfer, blood transfusion, or organ transplantation (2, 3).

Primary infection is usually asymptomatic in immunocompetent individuals. However, nearly 10% of infected individuals show symptoms that are especially characterized by the self-limiting mononucleosis-like syndrome (4). In patients suffering from immunodeficiency or hematological disorders, CMV infection can cause substantial morbidity and mortality due to the virus dissemination to multiple organs as a result of uncontrolled viral replication (5, 6). In these patients, the possibility of CMV transmission through blood transfusion remains a constant concern.

The prevalence of CMV varies globally, with the rates reaching 100% in developing countries (7). Prevalence rates of 41.9, 74.4, and 50.4% have been reported in France, Croatia, and the United States, respectively (8–10). In Brazil, there are few studies regarding the epidemiology of CMV infection. Previous reports have demonstrated different prevalence rates of CMV infection in Santa Catarina (96.4%), Rio de Janeiro (78.7%), and São Paulo (84.8%) (11, 12). Increasing CMV prevalence was also associated with kidney transplants in northern Brazil (1, 13).

Although epidemiological studies describing CMV infection in patients with hematological diseases are scarce, elevated prevalence rates have been reported in patients with thalassemia (95.9%) and hematological malignancies (75.5%) (14, 15). In the state of Bahia, Brazil, patients with different hematological diseases also showed elevated seroprevalence for CMV infection (89.4%) (16). This infection might produce a broad impact on the prognosis of these patients. Previous reports have indicated the association between CMV infection and the development of hematological disorders (17, 18). For example, cytopenia, which is usually found in patients submitted to hematopoietic stem cell transplant, is frequently linked to CMV infection (19).

The studies mentioned above demonstrate the importance of the epidemiological surveillance of CMV infection to the clinical management of patients suffering hematological diseases. Therefore, the present study aimed to describe the epidemiological profile of the CMV infection among patients with hematological diseases from the western Brazilian Amazon.

## Materials and Methods

### Ethical Approval

This study was approved by the Human Research Ethics Committee of the Hematology and Hemotherapy Hospital Foundation of Amazonas (approval number: 1.994.410). We ensured confidentiality to all participants, as well as the right to refuse to answer questions that could cause discomfort during the study. Patients submitted to anti-viral treatment during the research period were excluded from the study to avoid any confounding bias.

### Study Population

From December 2016 to August 2017, we randomly recruited 323 patients from attendees at the outpatient clinic of the Hemotherapy and Hematology Hospital Foundation of Amazonas—HEMOAM. Only patients with a confirmed diagnosis for hematological diseases were eligible to participate in this study. A standardized interviewer-administered questionnaire was used to obtain information on sociodemographic and risk factor variables. Individuals of either sex and different ethnicities who were aged from 1 to 92 years were selected.

### CMV Infection Diagnosis

Serum samples from the study population were tested for CMV IgM and IgG antibodies (Abs) through an enzyme-linked immunosorbent assay, which was performed according to the manufacturer's recommendations (Serion ELISA classic, Serion GmbH, Germany). The optical density (OD) was measured with a spectrophotometer using a 405 nm filter, and the test positivity was determined according to the cut-off formula indicated by the manufacturer. To estimate the cut-off ranges, the mean value of the OD of the positive controls was multiplied by the numerical data from the quality control certificate (OD = 0.600 × positive control mean for upper cut-off; OD = 0.350 × positive control mean for lower cut-off).

To differentiate primary infection from recurrent infection, serum samples from patients who were positive for IgM were submitted to the IgG avidity test. For this assay, the same commercial kit and protocol were used. However, one elution step was added with an 8 M urea solution as the agent antigen binding of low avidity. The tests were performed in duplicate, and the samples were tested with the addition of 8 M urea and without urea. The avidity index was calculated using the ratio of the samples' OD values that were treated with 8 M urea by the samples OD values that were untreated with urea, multiplied by 100 (IgG + 8 M urea/IgG × 100). Avidity index <45% was considered as indicative of a recent infection and >65% as a recurrent infection.

### Statistical Analysis

Descriptive statistical analysis was used to evaluate the sociodemographic variables by calculating measures of central tendency and dispersion. The results were categorized according to normality. The Odds Ratio (OR) analysis was employed to assess the association between socio-demographic factors and the type of hematological disease with susceptibility to CMV infection. OR values were estimated using Fisher's exact test. One-way ANOVA and Student's *t*-test were used to compare the OD values of CMV antibodies among the study population. The *F*-test was applied to evaluate the variances of the serum antibody levels. All statistical analyses were performed using Graphpad Prism v.5.0 and Biostat v.5.0. A value of *p* < 0.05 was considered significant.

## Results

### Prevalence of CMV Infection According to Hematological Disease and Blood Transfusion Rates

The presence of anti-CMV IgG Abs was detected in 295 patients (91.3%) (Table 1). From this number, 179 underwent two or more blood transfusions during a 1-year period. However, the association between transfusion and prevalence rates was not statically significant (*p* = 0.36).

**Table 1:** Overall prevalence of CMV infection and blood transfusion rates in patients with hematological diseases.

**Anti-CMV IgG**	**N (%)**	Transfusion rates^*^ (%)	**OR (95% CI)**	***p*** **-value**
Seropositive	295 (91.3)	179 (60.66%)^**^	0.8573 (0.382–1.922)	0.360
Seronegative	28 (8.7)	18 (72%)^***^	1.00	
Total	323	197 (61%)		

*OR, odds ratio; CI, confidence interval*.

* *Number of patients submitted to 2 or more transfusions during a 1-year period*.

** *Percentage taking into account the total number of seropositive individuals*.

*** *Percentage taking into account the total number of seronegative individuals*.

**Figure 1:**
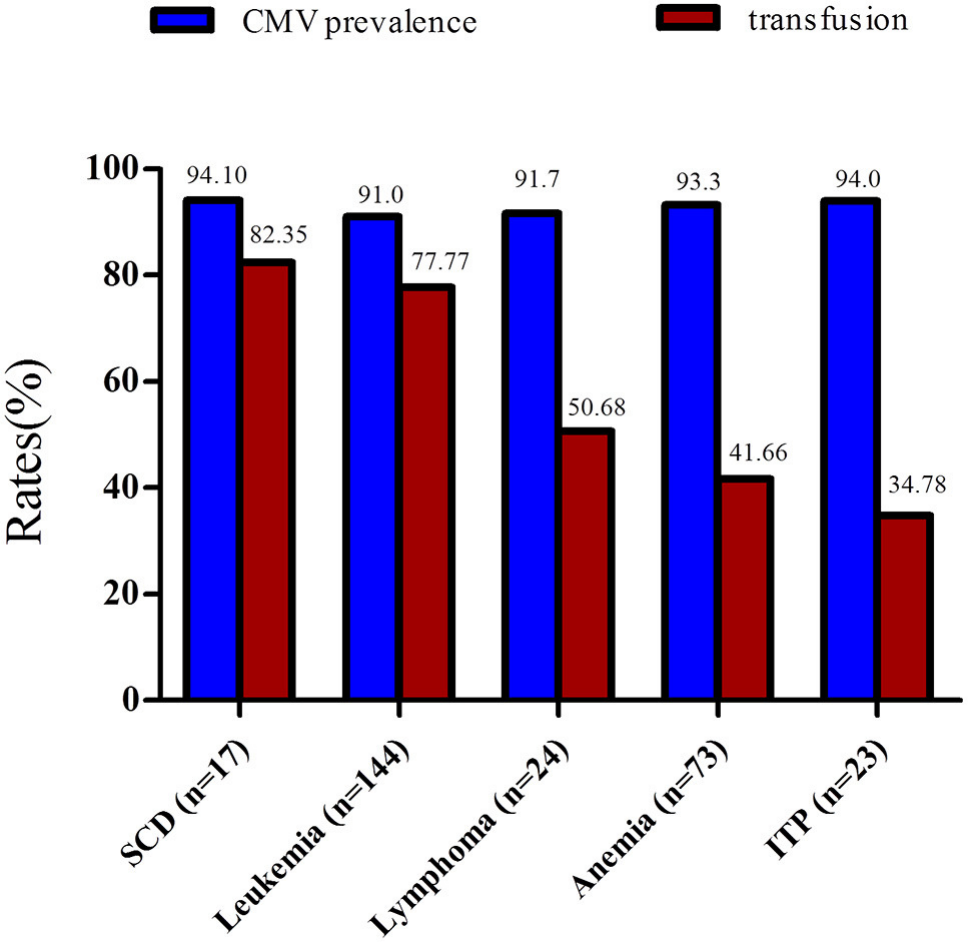
Rates of CMV prevalence and blood transfusions among patients with hematological diseases. All patients subject to this analysis were submitted to more than one blood transfusion during a 1-year period. ITP, immune thrombocytopenic purpura.

The prevalence of CMV infection according to the hematological disease and blood transfusion rates (1-year period) observed in this study are shown in Figure 1. Leukemia was the most prevalent hematological disease in the study population. Patients with leukemia showed CMV prevalence and blood transfusion rates of 93.3 and 77.7%, respectively. Anemia (different etiologies) was the second most frequent disease (Figure 1). A total of 37 patients (50.68%) suffered from severe anemia. These patients were often submitted to multiple blood transfusions per year.

Patients with lymphoma and immune thrombocytopenic purpura (ITP) exhibited a CMV prevalence of 91.7 and 94%, respectively (Table 2). Individuals with sickle cell anemia had the highest blood transfusion frequency (82.35%) and exhibited a CMV prevalence of 94.11%.

**Table 2:** Prevalence rates of CMV infection according to the type of hematological disease.

**Hematological diseases**	***N***	**CMV-positive *N* (%)**	**CMV-negative *N* (%)**	**OR (95% CI)**	***p*** **-Value**
Anemia (aplastic, sickle cell, hemolytic, and others)	90	84 (93.3)	6 (6.7)	1.45 (0.57–3.72)	0.565
Platelet diseases	39	37 (94.9)	2 (5.1)	1.86 (0.42–8.18)	0.592
Spherocytosis	2		2 (100)		
Hemophilia	9	9 (100)			
Hemoglobinopathies	3	3 (100)			
Leukemia	144	133 (92.4)	11 (7.6)	1.03 (0.42–2.36)	0.934
AML	24	23 (95.8)	1 (4.2)		
CML	14	13 (93)	1 (7)		
ALL	100	91 (91)	9 (9)		
CLL	3	3 (100)			
ATL	3	3 (100)			
Lymphoma	24	22 (91.7)	2 (8,3)	1.04 (0.23–4.70)	0.751
Multiple myeloma	4	3 (75)	1 (25)		
Polycythemia	1		1 (100)		
Myelodysplastic syndrome	3	3 (100)			
Thalassemia	4	3 (75)	1 (25)		

*AML, acute myeloid leukemia; CML, chronic myeloid leukemia; ALL, acute lymphocytic leukemia; CLL, chronic lymphocytic leukemia; ATL, adult T-cell leukemia/lymphoma; OR, odds ratio; CI, confidence interval*.

### CMV Prevalence According to the Sociodemographic Characteristics of Study Population

Most of the patients declared themselves as brown-skinned (65.3%) and single (70.5%). The average age of the study population was 26 years (Table 3). The prevalence of CMV infection observed among brown and black-skinned patients was 92.3 and 91.3%, respectively, whereas in white-skinned patients the CMV prevalence was 89.9%. No statistically significant association was observed between seropositivity for CMV infection and ethnicity or marital status.

**Table 3:** Prevalence rates of CMV infection according to sociodemographic characteristics of the study population.

**Sociodemographic characteristics**	***N*** **(%)**	**Age range**	**CMV-positive *N* (%)**	**CMV-negative *N* (%)**	**OR (95% CI)**	***p*** **-Value**
**Ethnicity**						
Black	23 (7.1)	2–53	21 (91.3)	2 (8.7)	1.00	
White	89 (27.6)	1–92	80 (89.9)	9 (10.1)	0.79 (0.34–1.81)	0.728
Brown	211 (65.3)	1–90	194 (91.9)	17 (8.1)	1.24 (0.56–2.75)	0.742
**Family income**						
1 minimum wage	163 (50.5)	1–90	149 (91.4)	14 (8.6)	1.02 (0.47–2.22)	0.884
2–5 minimum wages	145 (44.9)	1–92	132 (91.0)	13 (9.0)	0.93 (0.43–2.03)	0.978
6–9 minimum wages	13 (4.0)	7–67	12 (92.3)	1 (7.7)	1.00	
More than 10 minimum wages	2 (0.6)	48–55	2 (100.0)			
**Level of schooling**						
Illiterate^*^	23 (7.3)	1–92	22 (95.7)	1 (4.3)	2.13 (0.27–16.50)	0.729
Literate^**^	29 (9.2)	4–90	27 (93.1)	2 (6.9)	1.27 (0.28–5.72)	0.972
Elementary school	38 (12.1)	6–15	28 (73.7)	10 (26.3)	0.17 (0.07–0.42)	**<0.0001**
Incomplete middle school	7 (2.2)	12–14	6 (85.7)	1 (14.3)	0.54 (0.06–4.71)	0.900
Complete middle school	82 (26.1)	7–78	77 (93.9)	5 (6.1)	1.00	
Incomplete high school	1 (0.3)	16	1 (100.0)			
Complete high school	69 (22.0)	16–77	66 (95.7)	3 (4.3)	2.38 (0.69–8.24)	0.246
Undergraduate	35 (11.1)	19–67	33 (94.3)	2 (5.7)	1.60 (0.36–7.12)	0.766
Not Informed	30 (9.6)	3–90	27 (90.0)	3 (10.0)		
**Occupation**						
Student	94 (48.2)	5–33	79 (84.0)	15 (16.0)	0.11 (0.03–0.51)	**0.002**
Housewife	41 (21.0)	22–90	39 (95.1)	2 (4.9)	1.00	
Retired	17 (8.7)	50–92	17 (100.0)			
Unemployed	37 (19.0)	16–87	37 (100.0)			
Not informed	6 (3.1)	20–28	6 (100.0)			
**Condom usage**						
Always	61 (30.8)	16–60	58 (95.1)	3 (4.9)	1.00	
Intermittent	15 (7.6)	17–64	15 (100.0)			
Never	111 (56.1)	16–90	105 (94.6)	6 (5.4)	0.72 (0.17–2.97)	0.913
Not informed	11 (5.6)	16–90	11 (100.0)			
**Awareness of CMV infection**						
None	304 (94.1)	1–92	276 (90.8)	28 (9.2)		
Little	17 (5.3)	5–56	17 (100.0)			
Not informed	2 (0.6)	17–39	2 (100.0)			

*OR, Odds Ratio; CI, Confidence Interval*.

* *People able to read and write*.

** *People unable to read and write*.

*All individuals in both the groups literate and illiterate were not attending school. Brazilian national minimum monthly wage: approximately $192,00 US dollars*.

*Bold values highlight the signficance*.

The study population was comprised of individuals with low levels of education and low purchasing power. The majority of patients (50.4%) was from families earning a minimum wage (approximately $192,00 US dollars in Brazil). Individuals with middle-level education (26.1%), complete high school (22%), or elementary school (20.7%) were more frequent in the study population. Individuals with elementary education showed decreased susceptibility to CMV infection (*p* = 0.0002). The association between CMV infection and the occupation of students was also statistically significant (*p* = 0.005), suggesting that students are less susceptible to CMV infection. No correlation was observed between condom usage and positivity for CMV infection. An overwhelming number of patients did not know about CMV infection (94%), especially regarding transmission and prevention (Table 3).

### CMV Serological Profile of the Study Population

**Figure 2:**
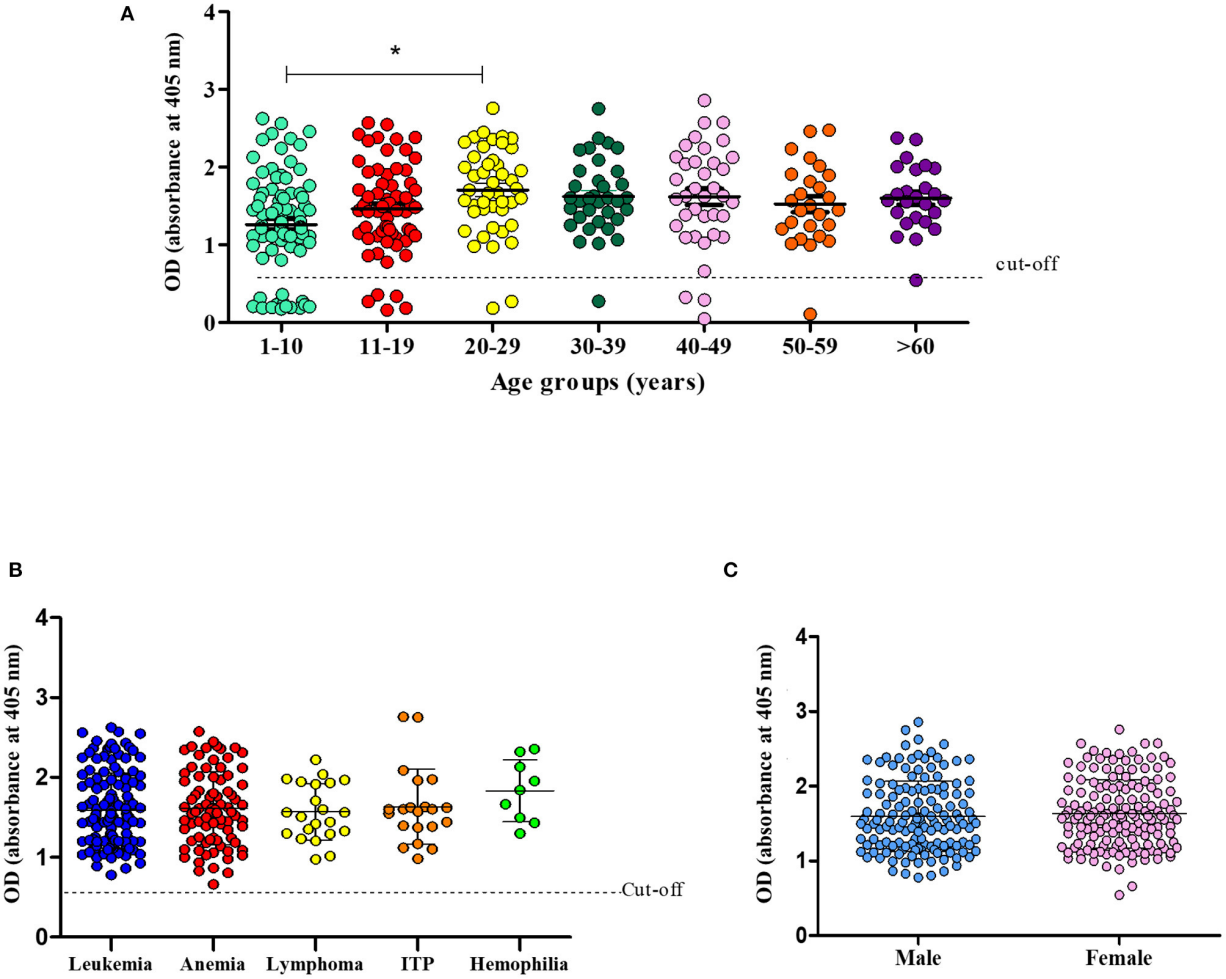
Prevalence of CMV infection according to age **(A)**. Number of CMV-infected patients according to hematological disease **(B)** and sex **(C)** (*p = 0.02, One-way ANOVA and Student's *t*-test).

The difference in prevalence observed between the age groups 1–10 and 20–29 was statistically significant (*p* = 0.02) (Figure 2A). However, when the prevalence was stratified according to the type of hematological disease, no significance was found (Figure 2B). A sex-based analysis showed similar prevalence rates (*p* = 0.750) between women (92.1%) and men (90.5%) (Figure 2C).

**Figure 3:**
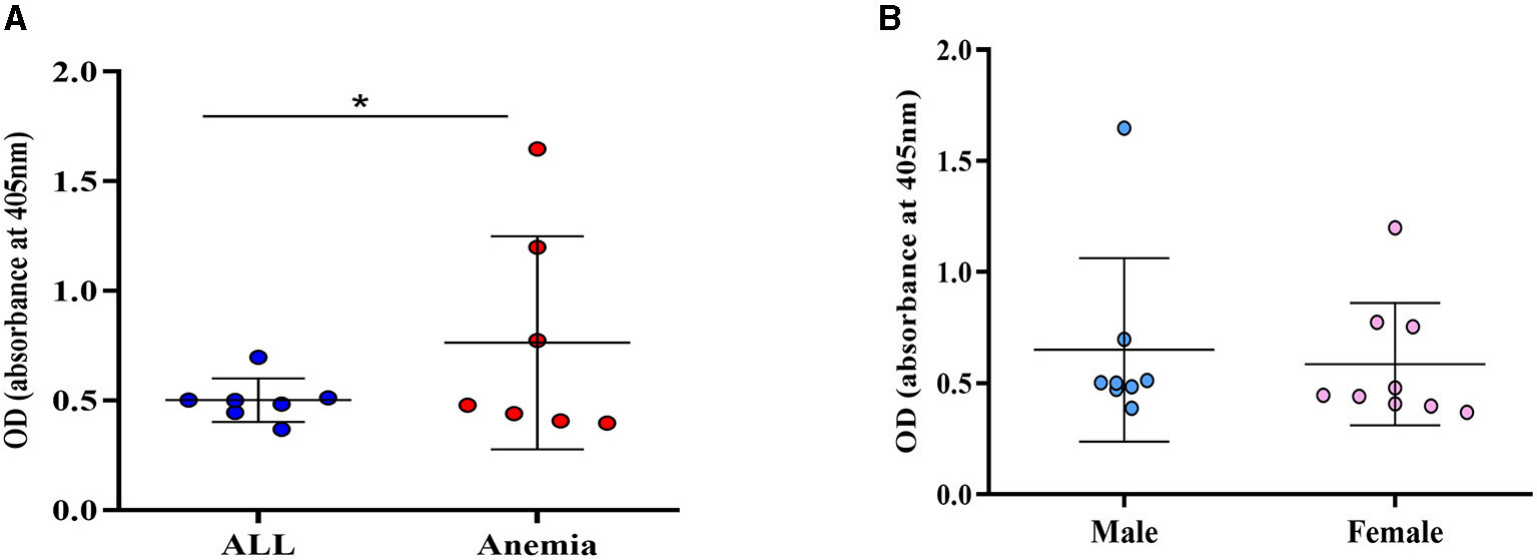
Variance of CMV IgM Ab positivity. **(A)** OD levels between patients with ALL and anemia; **(B)** OD-values according to sex; *p = 0.001 (*F*-test).

The occurrence of active CMV infection was assessed through the detection of serum IgM Ab. A seropositivity of 5.3% (17/323) for anti-CMV IgM Ab was observed among the study population. Anti-CMV-IgM positive patients were carriers of anemia of different etiologies (*n* = 7), ALL (*n* = 7), lymphoma (*n* = 2), and thrombocytopenia (*n* = 1). The variance of the CMV-IgM Ab positivity observed between patients with anemia and patients with ALL was statistically significant (*p* = 0.01) (Figure 3A). No statistical significance was observed between IgM-Ab positivity and sex (*p* = 0.45) (Figure 3B).

**Figure 4:**
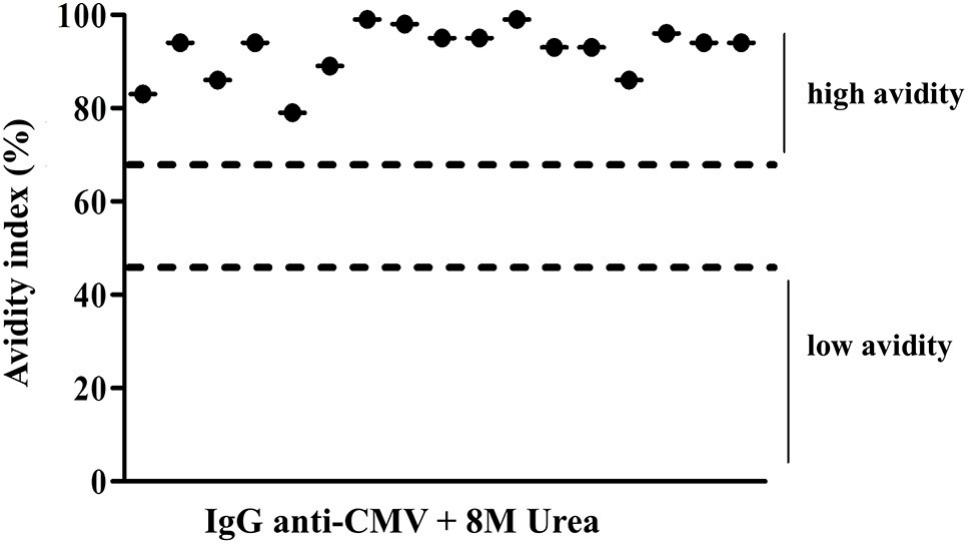
Avidity index values of IgG CMV levels of positive patients to IgM CMV. Low avidity represented values <45% and high avidity represented values >65%.

The IgG avidity test revealed that all CMV active infections resulted from a recurrence of the infection since the individuals showed an avidity index >60% (Figure 4).

## Discussion

Despite the fact that CMV infection is widespread all over the world, the epidemiological surveillance of this virus is still neglected (20). Raising awareness regarding the dissemination of CMV in Brazil is imperative in order to combat the infection and for the clinical management of immunocompromised patients, especially patients with hematological diseases.

Our findings demonstrated that the CMV infection is highly prevalent among patients from the western Brazilian Amazon with hematological diseases. The study population showed a prevalence rate (91%) that is higher than the one observed (67.6%) in the city of Manaus but, in both cases, young adults were more susceptible to CMV infection (21). Likewise, a study carried out at the Hemotherapy and Hematology Foundation in the state of Bahia (HEMOBA), in Brazil, observed an increased prevalence rate of CMV infection (89.4%) in patients with different hematological diseases (16). Preeminent seropositivity for CMV infection was also observed in patients from Iran with thalassemia (95.9%) and patients from China with idiopathic thrombocytopenic purpura (86.4%) (15, 22). Our results reveal a wide CMV circulation in the study population. The present study is a pioneer in describing the epidemiology of CMV infection in patients suffering from hematological diseases in the western Brazilian Amazon.

Cytomegalovirus infection tends to be more frequent in women than in men. This situation probably occurs due to the greater susceptibility of women to transmission via sexual contact and because women generally spend more time taking care of children (working in daycare centers or at home), as suggested by some studies (5, 20). In this study, we did not find any correlation between the sex of the study population and susceptibility to CMV infection. Similarly, no difference in prevalence rates was observed in white and non-white-skinned individuals, even though positivity for CMV infection has been previously found to be 30% higher in non-white-skinned people (20). Our findings also demonstrated that having elementary education and being a student was directly associated with low susceptibility to CMV infection. However, the low level of education, inadequate sanitary conditions, cultural aspects, and families with a great number of individuals have already been described as the main factors that are attributed to elevated CMV prevalence rates (10, 23). Similarly, previous findings have shown CMV prevalence ranging from 75 to 97.7% among university students from the Middle East (24, 25). Nevertheless, the majority of students of the study population were very young, which may explain the low susceptibility to CMV infection observed in these groups since the prevalence rates were more prominent among older people. In the present study, nearly half of the patients (43.7%) declared no condom usage during sexual intercourse, but we did not find any correlation between this factor and the susceptibility for CMV infection.

Our results demonstrated that the prevalence of CMV infection was greater in patients with anemia of different etiologies (93.3%), platelet diseases (94.9%), lymphoma (91.7%), and leukemia (91%). Increased prevalence of CMV infection has been previously described in patients with aplastic anemia, lymphoma, or leukemia (26–28). In some cases, the development of these diseases and the morbimortality of patients were related to CMV infection (26, 28, 29). Epidemiological studies showing CMV infection prevalence in patients with platelet disease are scarce, but the correlation between the development of thrombocytopenia and CMV infection was also reported. Although this is not a typical situation, CMV infection may lead to thrombocytopenia or thrombocytopenic purpura in healthy children during the neonatal period (30). In the present study, we did not analyze the association between CMV infection and the development of these diseases, nor its connection with the enhancement of patients' morbimortality. However, the epidemiological surveillance of CMV infection in patients with hematological diseases provided by this study may be an important tool for improving their clinical management.

Detection of serum anti-CMV IgM Abs may indicate a recent infection or a recurrent infection (reactivation/reinfection) (31–33). Elevated IgM and low IgG Abs titers suggest primary infection rather than reactivation or reinfection (33). All 17 active infections observed in our study resulted from the recurrent infection since the patients presented high avidity CMV IgG. Positivity rates for anti-CMV IgM Abs vary according to population, culture, and region. A study carried out with women of reproductive age from the United States observed 3.0% positivity for anti-CMV IgM Abs (34). Pregnant women from Ireland showed 5.9% positivity for anti-CMV IgM Abs, whereas among patients from Croatia undergoing hemodialysis the rate observed was 2.3% (9, 35). In Brazil, 1.9% of blood donors from the southern region displayed anti-CMV IgM positivity (11). The present study found seropositivity of 5.3% for anti-CMV IgM Abs in the study population, which is higher than most of the rates described elsewhere. These findings suggest that hematological diseases may influence the recurrence of CMV infection.

In the context of hematological diseases, recurrent CMV infection has been typically associated with the immunosuppression caused by therapeutic schemes. An elevated proportion of CMV reactivation (84.6%) was observed in children with hemoglobinopathies submitted to hematopoietic stem cell transplantation and alemtuzumab treatment (36). In the present study, the clinical records of the study population were not available for us to search for possible links between treatment and the recurrence of CMV infection.

Most patients that were positive for anti-CMV IgM Abs suffered from ALL or anemia (various etiologies). Cytomegalovirus reactivation is considered to be elevated in individuals with leukemia. Increased rates of CMV reactivation were equally noted in patients from India (11.3%) and Iraq (12%) who suffer from leukemia (37, 38). Another study observed CMV reactivation in 66% of patients with chronic lymphocytic leukemia, after alemtuzumab therapy (39). These studies indicate that leukemia increases the risk of recurrent CMV infection by unknown mechanisms. However, it has been recognized that the natural killer (NK) cells are the key factor in combatting CMV infection (40).

Natural killer cell abnormality or deficiency is a risk factor for CMV reactivation (41). This condition could explain the occurrence of elevated CMV reactivation among patients with ALL described in this study and elsewhere, since NK cell abnormalities have already been reported in these patients (42). In this study, we did not assess the phenotype of NK cells in patients who were seropositive for anti-CMV IgM in order to verify this correlation. However, our findings raise the following questions: Are the patients with ALL or anemia more prone to CMV recurrence? Are recurrence rates associated with immunological suppression conditions that are inflicted by ALL or anemia? A longitudinal study with a larger sample size is required in order to answer these questions.

Taken all together, our findings bring a new understanding of the epidemiological profile of CMV infection in patients from the northern region of Brazil who suffer from hematological diseases. The data presented here may be a starting point for prospective studies, especially those that seek to elucidate the negative prognostic impact of CMV infection in patients with hematological diseases.

## Conclusions

In summary, this study presents new findings on CMV infection epidemiology by serologically characterizing this infection in patients from the western Brazilian Amazon region with hematological diseases. Cytomegalovirus infection is highly prevalent among the study population and recurrent infection is more frequent in patients with ALL or anemia. Age, level of education, and occupation are factors feasibly correlated with the CMV infection among the study population. According to our findings, blood transfusions were not associated with increased susceptibility to CMV infection in the study population.

These results may contribute to the surveillance of CMV infection performed by the Brazilian public healthcare system since no such system for surveillance of CMV currently exists and thus could provide a new basis for more comprehensive prospective studies involving patients with hematological diseases.

## Data Availability Statement

The original contributions presented in the study are included in the article/supplementary material, further inquiries can be directed to the corresponding author/s.

## Ethics Statement

The studies involving human participants were reviewed and approved by the Human Research Ethics Committee of the Hematology and Hemotherapy Hospital Foundation of Amazonas (Approval Number: 1.994.410). Written informed consent to participate in this study was provided by the participants' legal guardian/next of kin.

## Author Contributions

GP and JM: conceptualization. JM, RC, and CC: methodology and investigation. GP, JM, and RP-S: validation. GP, AB, JM, and RP-S: formal analysis. GP, RP-S, and JM: resources. GP and AB: data curation. JM and RP-S: writing—original draft preparation. GP and RP-S: writing—review and editing. GP: supervision, project administration, and funding acquisition. All authors have read and agreed to the published version of the manuscript.

## Funding

This study was financed in part by the Coordenação de Aperfeiçoamento de Pessoal de Nível Superior- Brasil (CAPES)- Finance code PROCAD AMAZÔNIA 88881.200581/201801 and Fundação de Amparo à Pesquisa do Estado do Amazonas (FAPEAM—Pró-Estado Program).

## Conflict of Interest

The authors declare that the research was conducted in the absence of any commercial or financial relationships that could be construed as a potential conflict of interest.

## Publisher's Note

All claims expressed in this article are solely those of the authors and do not necessarily represent those of their affiliated organizations, or those of the publisher, the editors and the reviewers. Any product that may be evaluated in this article, or claim that may be made by its manufacturer, is not guaranteed or endorsed by the publisher.
